# Supplementary material for: CD4 expression in effector T cells depends on DNA demethylation over a developmentally established stimulus-responsive element
Source: Nat Commun. 2022 Mar 18;13:1477. doi: 10.1038/s41467-022-28914-4 (PMC8933563; doi:10.1038/s41467-022-28914-4)
Supplement: Supplementary file 1 — Supplementary Information [file 41467_2022_28914_MOESM1_ESM.pdf]

SUPPLEMENTARY INFORMATION

CD4 Expression in Effector T Cells Depends on DNA Demethylation over a Developmentally Established Stimulus-responsive Element

Athmane Teghanemt[1,2], Priyanjali Pulipati[1,2,#], Kara Misel-Wuchter [1,3,#], Kenneth Day[4], Matthew S. Yorek[1,2], Ren Yi[5], Henry L Keen[6], Christy Au[7], Thorsten Maretzky[1,2], Prajwal Gurung[1,2], Dan R. Littman[7,8], Priya D. Issuree[1,2,3,9,*].

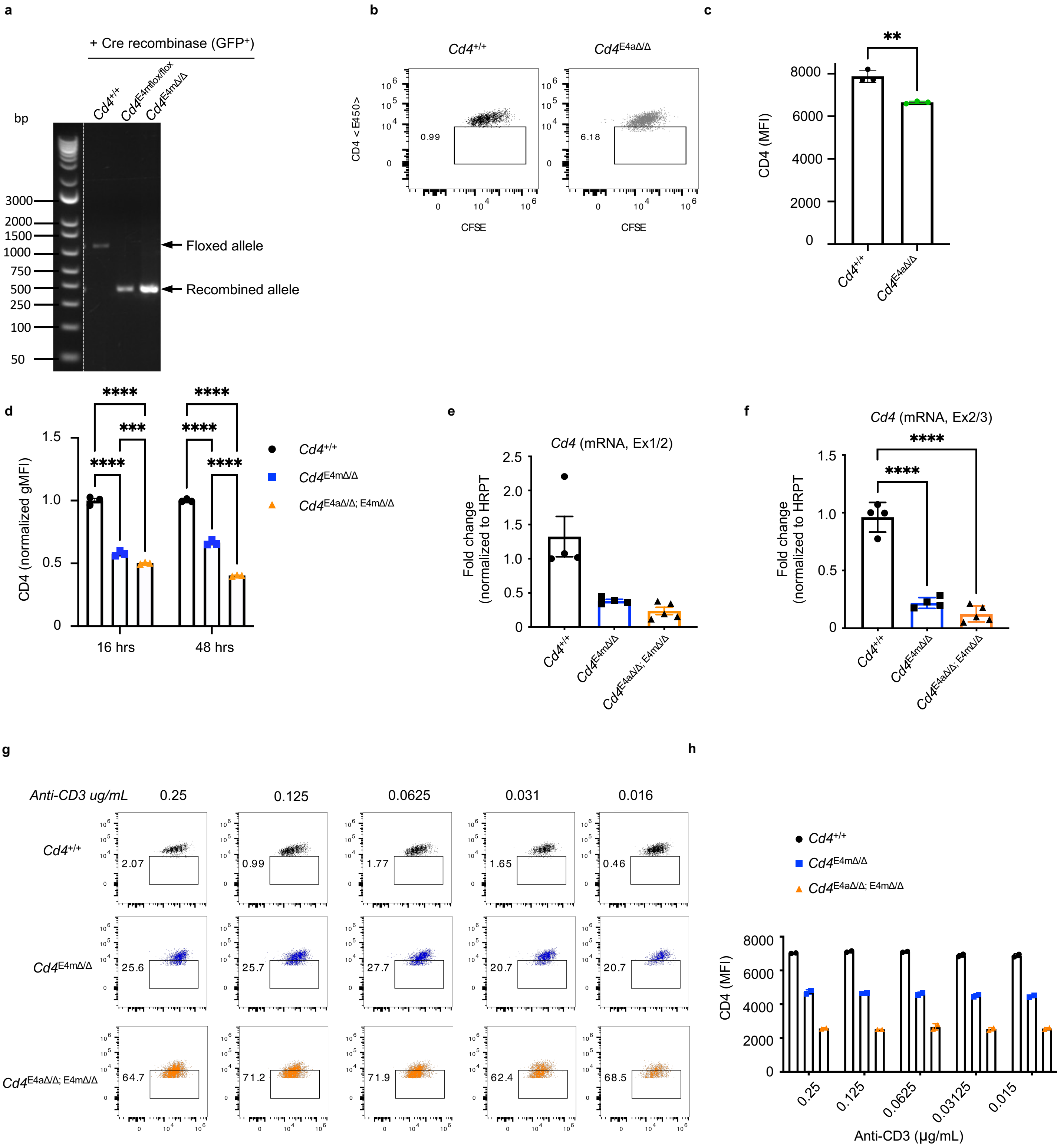

Supplementary Figure 1

SUPPLEMENTARY FIGURE LEGENDS

Supplementary Fig.1: E4a modulates *Cd4* expression in effector T cells in a partially redundant manner with E4m. **a**, DNA electrophoresis gel showing excision of the E4m allele after Cre-recombinase expression. Transduced T cells (GFP^+) were FACS-sorted 96hrs post-transduction and genomic DNA was isolated for PCR analysis. DNA from $Cd4^{E4m\Delta/\Delta}$ was used as a positive control. **b**, Dot Plot showing CD4 expression and CFSE dilution on control and $Cd4^{E4a\Delta/\Delta}$ CD4 T cells, 72hrs post activation with anti-CD3 and CD28. Experiment is representative of >3 experiments. **c**, Bar graph showing CD4 MFI on *in vitro* activated T cells from control or $Cd4^{E4a\Delta/\Delta}$ mice analyzed 72 hrs post activation. Cells were gated at equivalent cell division cycles. (n=3) and data shown is representative > 3 experiments and expressed as mean ± SD. ** p=0.0019 (unpaired two-tailed t test). **d**, Bar graph showing CD4 gMFI on *in vitro* activated T cells from indicated genotypes analyzed 16 hrs and 48 hrs post activation. n=3 independent samples. Data shown is a summary of 2 experiments and expressed as mean ± SEM. ***p=0.0009, ****p<0.0001 (Two-Way ANOVA and Bonferroni test). **e**, CD4 mRNA expression (exon 1-2) in *in vitro* activated CD4 T cells with indicated genotypes. RNA was isolated 96hrs post activation. (n=4 for group 1 and 2; n=5 group 3). Data is expressed as mean ± SEM. (One-Way ANOVA with Bonferroni multiple comparison test). **f**, CD4 mRNA expression (exon 2-3) in *in vitro* activated CD4 T cells with indicated genotypes. RNA was isolated 96hrs post activation. (n=4 for group 1 and 2; n=5 group 3). Data is expressed as mean ± SEM.***p<0.0001 (One-Way ANOVA with Dunnett's Multiple comparison test). **g**, FACS dot plots showing CD4 expression and CFSE dilution at 72hrs on CD4 T cells following activation with indicated doses of anti-CD3 and 1ug/mL anti-CD28. Data is representative of two independent experiments. **h**, CD4 gMFI expression on CD4 T

cells 72hrs post activation with indicated concentrations of anti-CD3 and 1ug/mL anti-CD28. Cells were gated on equivalent cell division numbers and data shown is representative of two independent experiments (n=2/group).

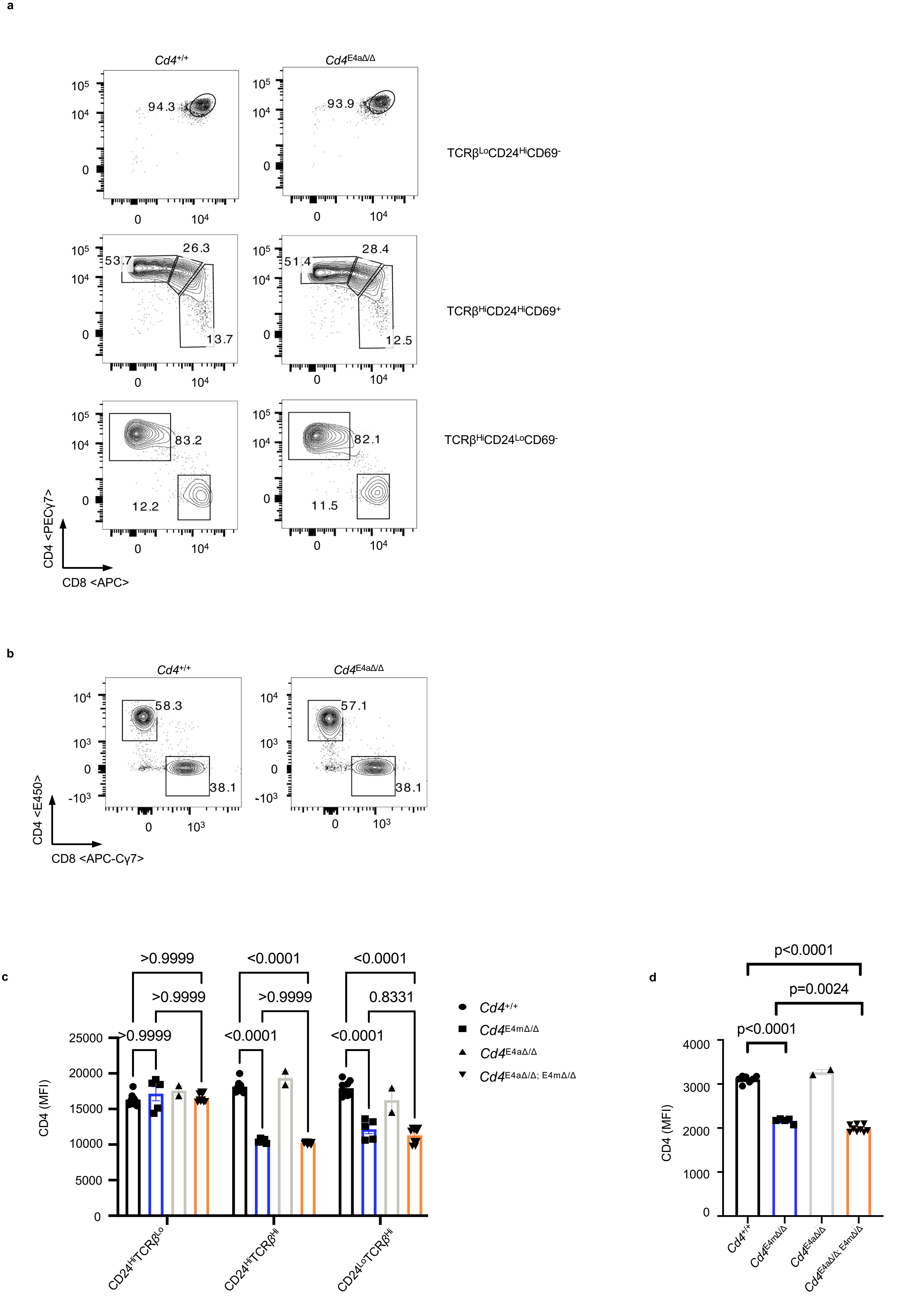

Supplementary Figure 2

Supplementary Fig. 2: E4a is a stimulus-responsive *cis*-regulatory element licensed during development. **a**, FACS contour data plots showing pre-selected TCRβ^{Lo}CD69^{-}CD24^{hi}DP (top panel), recently selected TCRβ^{hi}CD69^{+}CD24^{hi} (middle panel) and mature TCRβ^{hi}CD69^{-}CD24^{lo} T cell populations (bottom panel) in the thymus of mice with indicated genotypes. Data is representative of >3 experiments. **b**, FACS contour data plots showing T cell populations among TCRβ+ T cells in the spleen/LN of $Cd4^{+/+}$ and $Cd4^{E4a\Delta/\Delta}$ mice. Data is representative of >3 experiments. **c**, Bar graph quantifying CD4 MFI on different CD4 T cell populations from the thymus from mice with the indicated genotypes. Data is expressed as mean ± SEM (n = 8 for $Cd4^{+/+}$; n=5 for $Cd4^{E4m\Delta/\Delta}$;n=2 for $Cd4^{E4a\Delta/\Delta}$; n=10 for $Cd4^{E4a\Delta/\Delta\ E4m\Delta/\Delta}$) p-values are indicated on graph (Two-way ANOVA and with Bonferroni multiple comparison test). **d**, Bar graph quantifying CD4 MFI on TCRβ+ CD4 T cell populations from the spleen/LN of mice with the indicated genotypes. (n = 7 for $Cd4^{+/++/+}$; n=5 for $Cd4^{E4m\Delta/\Delta}$; n=2 for $Cd4^{E4a\Delta/\Delta}$; n=9 for $Cd4^{E4a\Delta/\Delta\ E4m\Delta/\Delta}$) and a summary of two independent experiments .Data is expressed as mean ± SEM. p-values are indicated on graphs. (One-way ANOVA with Bonferroni multiple comparison test)

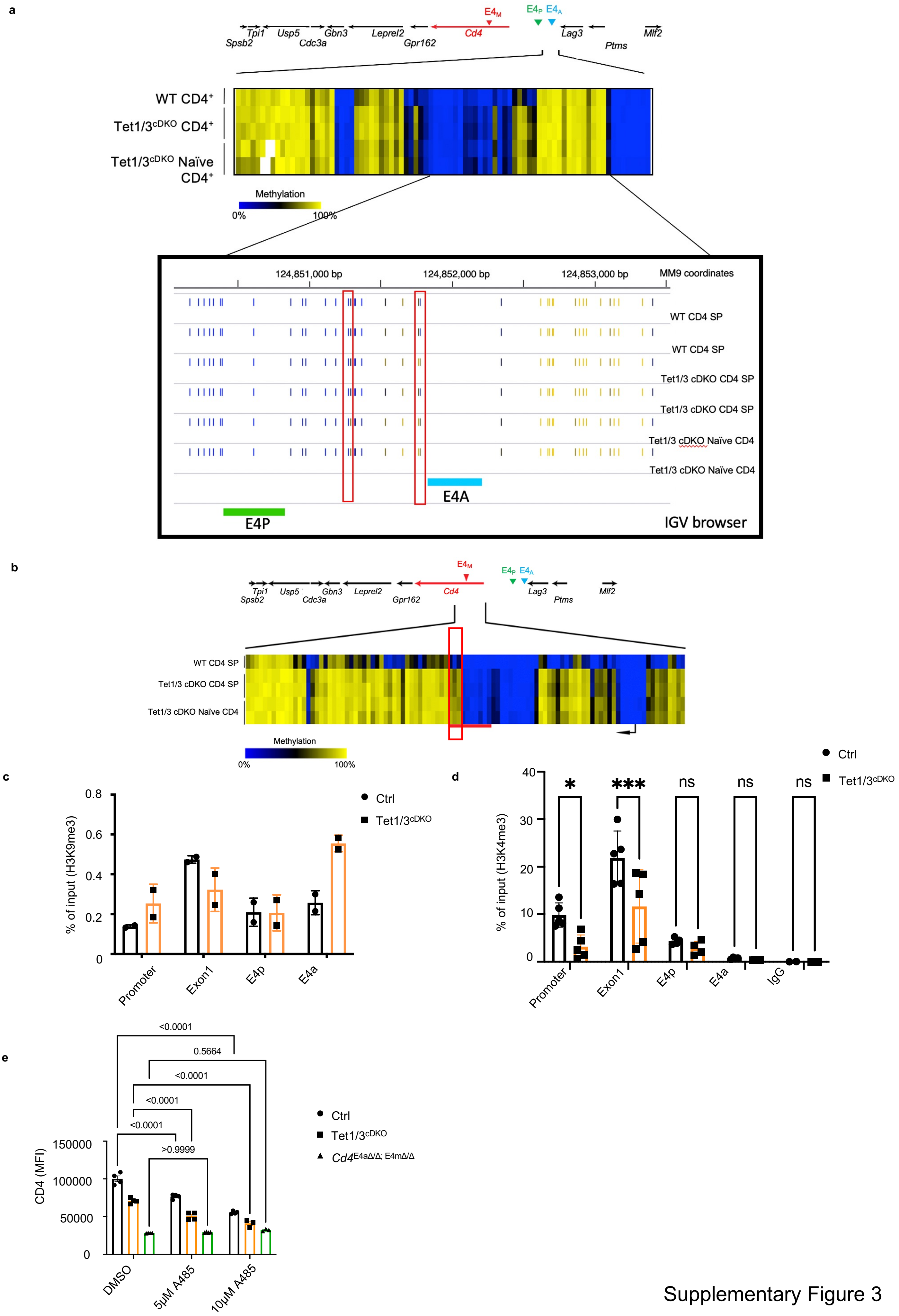

Supplementary Figure 3

Supplementary Fig. 3: Lack of DNA demethylation during development affects the function of E4m/E4a in effector CD4-lineage T cells. **a**, Heatmap (Top) and IGV snapshot (bottom) of the *Cd4* locus depicting individual CpGs with MM9 coordinates captured by CATCH-Seq in regions proximal to E4p (green) and E4a (blue). Differential CpGs flanking E4a are highlighted in red boxes. **b**, Heatmap depicting percent CpG methylation in control $CD4^+$ (Tet1/$3^{flox/flox}$), Tet1/3^{cDKO} $CD4^+$ mature thymocytes and Tet1/3^{cDKO} $CD4^+$ naïve peripheral T cells for CpGs from +6200 to −669 relative to the *Cd4* TSS (Chr6:124832027–124838896; mm9). A red line underlines CpGs in E4m (indicated by the gap in the mutant mice) and a black arrow indicates the *Cd4* TSS. CATCH-seq was performed on genomic DNA from sorted populations of $TCR\beta^{hi}CD24^{lo}CD69^{-}CD4^{+}CD8^{-}$ thymocytes or $CD4^{+}TCR\beta^{+}CD62L^{hi}$ $CD44^{-}$ T cells from LN/Spleen. Replicates are from 2 independent mice. **c**, Histone H3K9me3 modifications assessed by ChIP-qPCR in sorted naïve CD4 T cells activated *in vitro* for 120hrs. (n=2) and representative of 2 independent experiments. **d**, Histone H3K4me3 modifications assessed by ChIP-qPCR in sorted naïve CD4 T cells activated *in vitro* for 120hrs. (n= 5) and data is a summary of 2 independent experiments. Data is expressed as mean ± SEM. *p=0.0448, ***p=0.0009, ns= not significant, p>0.9999 (Two-Way ANOVA with Bonferroni multiple comparison test). **e**, Bar graph quantifying CD4 gMFI on T cells from indicated genotypes treated with DMSO or A-485 and analyzed 72hrs post activation with anti-CD3/CD28 *in vitro*. Cells were treated with DMSO vehicle control or indicated doses of A-485 18 hrs post-activation. (n=3 or 4) and data is representative of 3 experiments. Data is expressed as mean ± SD (Two-way ANOVA and Tukey's multiple comparison test)

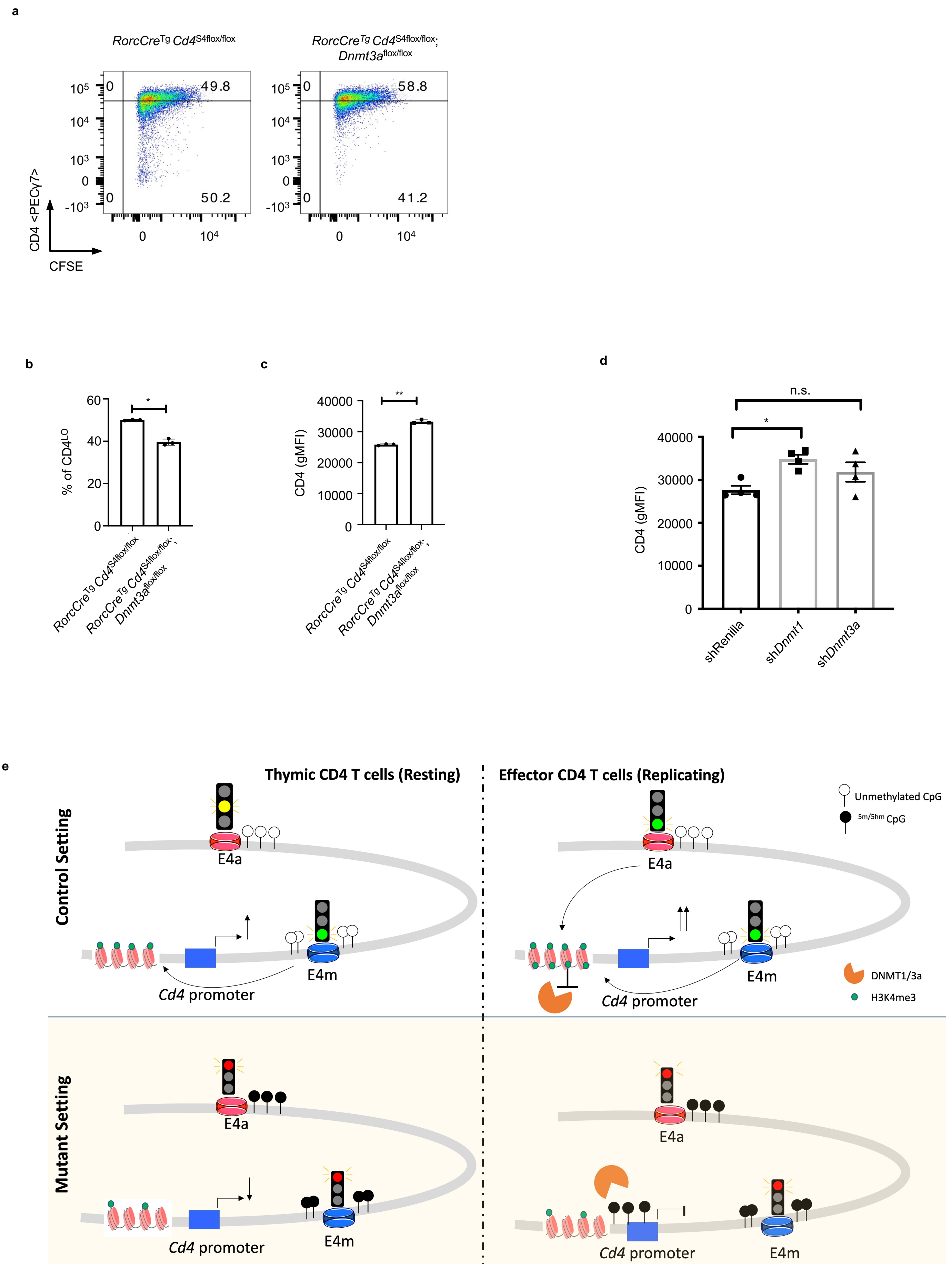

a
RorcCreTg Cd4S4flox/flox
RorcCreTg Cd4S4flox/flox; Dnmt3aflox/flox
49.8
50.2
58.8
41.2
CD4 <PECy7>
CFSE
b
% of CD4LO
c
CD4 (gMFI)
d
n.s.
shRenilla
shDnmt1
shDnmt3a
e
Thymic CD4 T cells (Resting)
Effector CD4 T cells (Replicating)
Control Setting
Mutant Setting
Unmethylated CpG
5m/5hm CpG
E4a
E4m
Cd4 promoter
DNMT1/3a
H3K4me3

Supplementary Fig. 4: Reduced enhancer activity as a result of DNA methylation leads to *Cd4* promoter silencing during replication of effector CD4^{+} T cells. a, FACS plot showing loss of CD4 expression 96hrs after *in vitro* proliferation of activated CD4^{+} T cells isolated from RorcCre^{Tg} *Cd4*$^{S4fl/fl}$ and *Rorc(t)*^{CreTg} *Cd4*$^{S4fl/fl}$ *Dnmt3a*$^{fl/fl}$ mice. Data is representative of 3 experiments **b,** Bar graph quantifying the % of CD4 T cells from indicated genotypes losing CD4 expression 96hrs after *in vitro* proliferation (n=3). Data is expressed as mean ± SD *p<0.0002 (unpaired two-tailed t test)**. c,** Bar graph quantifying CD4 gMFI 96hrs post *in vitro* activation. (n=3 independent samples and representative of 2 independent experiments). Data shown is expressed as mean ± SD **p<0.0001 (unpaired two-tailed t test)**. d,** Bar graph quantifying CD4 gMFI in activated T cells from *Rorc(t)*^{CreTg} *Cd4*$^{S4fl/fl}$ *Dnmt3a*$^{fl/fl}$ mice, 96hrs post transduction with an shRNA against Renilla (control), Dnmt1 or Dnmt3a. (n=4 independent samples and representative of 2 independent experiments). Data is expressed as mean ± SD. ns= not significant, p=0.1725; *p=0.02 (One-way ANOVA and Sidak's multiple comparison test)**. e,** Model depicting the importance of E4m and E4a enhancer activities and CD4 gene expression in resting CD4+ thymic T cells versus activated effector CD4 T cells. E4a and E4m enhancer activities are critical for maintaining H3K4me3 levels at the promoter during replication. A lack of enhancer activity (E4m/E4m doubly deficient T cells) or reduced E4a/E4m enhancer activities (Tet1/3-deficient T cells) leads to a gain of *de novo* methylation and suppression of promoter activity and ultimately the loss of CD4 gene expression.

a

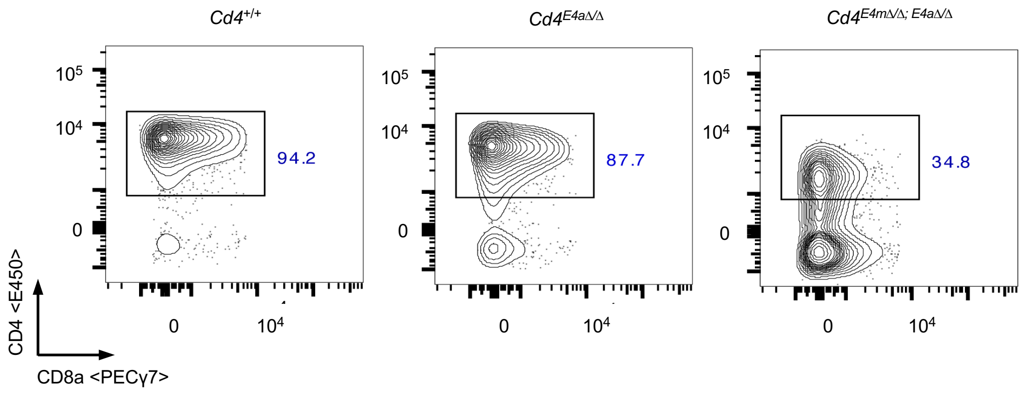

b

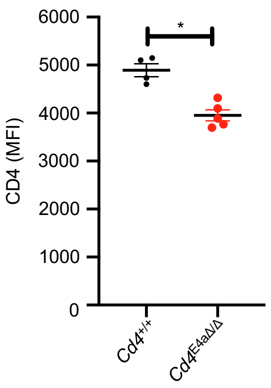

c

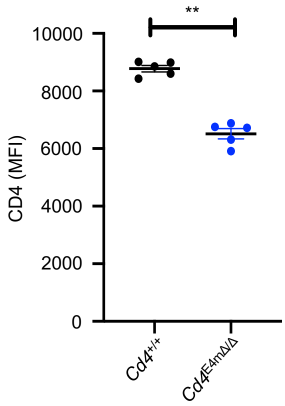

Supplementary Figure 5

Supplementary Fig. 5: Reduced CD4 expression in effector CD4 T cells impairs parasitic clearance during Leishmaniasis. a, FACS contour plots showing CD4 expression on cells gated on $CD11a^{+}CD44^{hi}$ T cells among $CD8^{-}$ $TCR\beta^{+}$ T cells. Cells were isolated from the dLNs of Leishmania infected mice at day 9 post infection. **b, c**, quantification of CD4 MFI on $CD11a^{+}CD44^{hi}$ T cells among $CD8^{-}$ $TCR\beta^{+}$ T cells from d9 infected mice.(n= 4 for $Cd4^{+/+}$; n=5 for $Cd4^{E4a\Delta/\Delta}$) Data is expressed as mean ± SEM *p=0.0159 (Mann-Whitney t- test) .

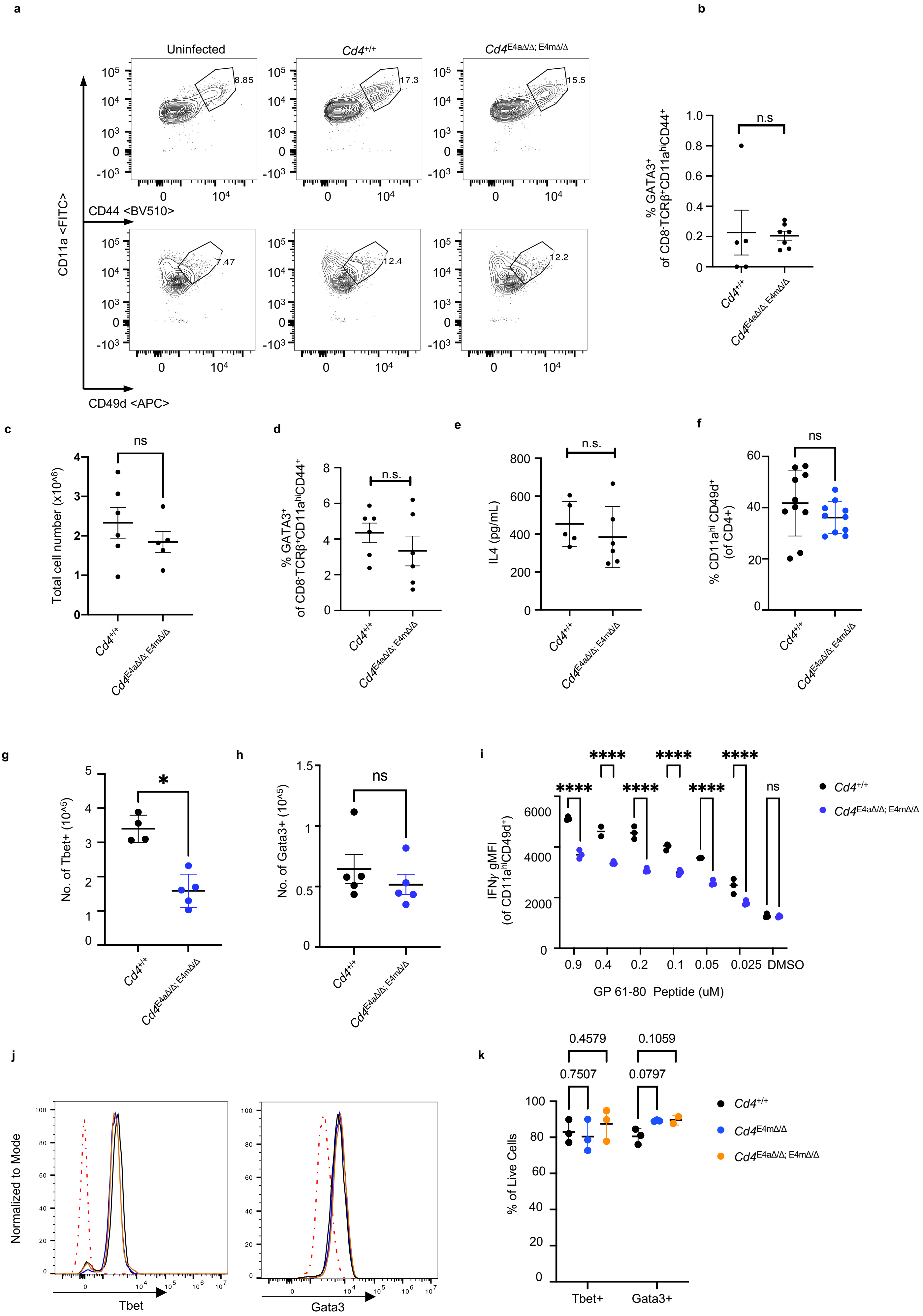

Supplementary Figure 6

Supplementary Fig. 6: Reduced CD4 expression impairs the differentiation of Th1 cells during Leishmaniasis. a, FACS contour plot depicting proportions of $CD11a^{hi}CD44^{+}$ T cells among $CD8^{-}$ $TCR\beta^{+}$ T cells (Top panel) or $CD11a^{hi}CD49d^{+}$ T cells among $CD8^{-}$ $TCR\beta^{+}$ T cells in the draining inguinal LNs of *L.major*-infected mice, 9 days post infection. Data is representative of > 3 independent experiments. **b,** Proportion of GATA3+ cells among $CD8^{-}$ $TCR\beta^{+}$ $CD11a^{hi}CD44^{+}$T cells from the popliteal dLNs of mice, 28 days p.i. (n=5 and 7 respectively) and is a summary of 2 independent experiments. Data is expressed as mean ± SEM. ns=not significant, p=0.4482 (Mann-Whitney test)**. c,** Absolute number of $CD8^{-}$ $TCR\beta^{+}$ T cells from the draining inguinal LNs of d28 *L.major*-infected mice. (n=6 and 5 respectively) Data is expressed as mean ± SEM. ns=not significant, p=0.4482 (Mann-Whitney test) **d,** Proportion of GATA3+ cells among $CD8^{-}$ $TCR\beta^{+}$ $CD11a^{hi}CD44^{+}$T cells from the popliteal LNs of mice, 9 days p.i. (n=6). Data is expressed as mean ± SEM and representative of 2 independent experiments; ns=not significant, p=0.5584 (Mann-Whitney test)**. e,** IL-4 levels in the supernatants from homogenized footpads of *L. major*–infected mice (n=5 and 6) on day 28 analyzed by multiplex ELISA. Data is expressed as mean ± SEM ns= not significant, p=0.3290 (Two-tailed Mann-Whitney *U* test). **f**, Proportions of $CD11a^{hi}CD44^{+}$ T cells among $CD8^{-}$ $TCR\beta^{+}$ T cells in spleen of LCMV-infected mice, 8 days p.i. (n=10). Data shown is mean ± SEM and is a summary of 2 independent experiments. ns=not significant, p=0.1655 (Two-tailed Mann-Whitney *U*-test). **g,h**, Proportion of Tbet+ or Gata3+ T cells among $CD8^{-}$ $TCR\beta^{+}$ $CD11a^{+}CD44^{hi}$T cells from in spleen of LCMV-infected mice, 8 days p.i. Data shown is a representative of 2 independent experiments with 6 mice/group. (Two-tailed Mann-Whitney *U* test)**. i.** IFNγ gMFI among TCRβ+ $CD8^{-}$ $CD11a^{hi}CD49d^{+}$ splenocytes of LCMV-infected mice, re-stimulated *in vitro* with varying doses of $GP_{61\text{-}80}$ peptide (n=3). ns=not significant, p>0.9999 ****p<0.0001 (Two-Way ANOVA with Sidak multiple comparison test).

j, Representative histogram plots showing expression levels of Tbet and Gata3 among sorted CD4+ T cells activated for 72hrs in the presence of polarizing cytokines. Dotted lines show staining under Th0 conditions (no polarizing cytokines). **k**, Quantification of data shown in **j**. (n=3) p-values shown on graph (Two-Way ANOVA with Dunnett's multiple comparison test) (Data is representative of 3 independent experiments.

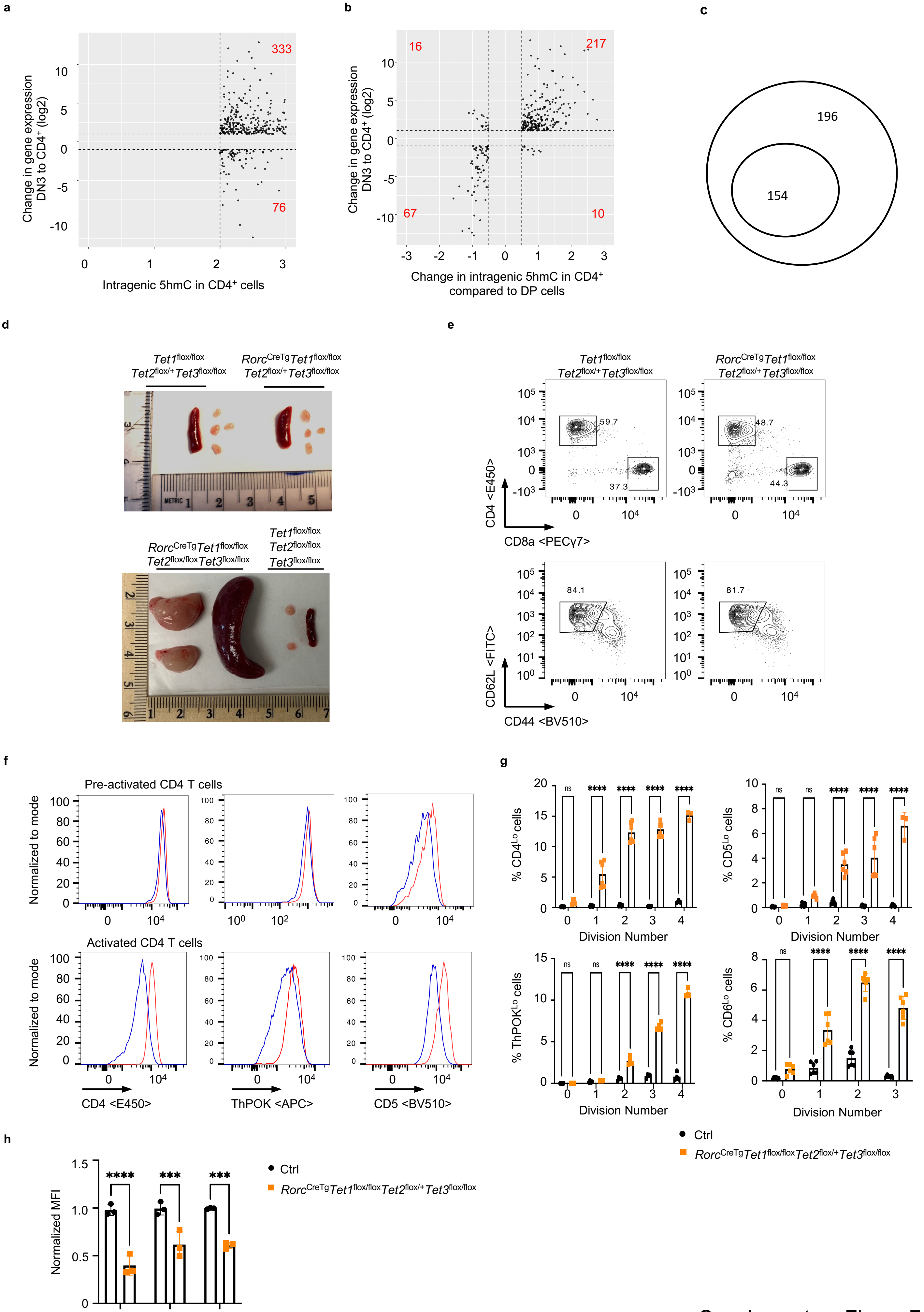

Supplementary Figure 7

Supplementary Fig. 7: TET-mediated demethylation during thymic development is critical for optimal gene function in effector T cells a, Scatter plot showing a fold change >2 in gene expression from DN3 to CD4+ thymic T cell differentiation (y-axis) versus presence of 5hmC in CD4+ T cells (log2 CMS-IP/Input >2). Number of genes in each quadrant is shown and depicted genes are listed in Supplementary Data 1. **b,** Scatter plot depicting change in gene expression from DN3 to CD4+ thymic T cell differentiation ((log2FoldChange) > 1) versus change in intragenic 5hmC in CD4+ compared to CD4+CD8+ DP thymic T cells. Genes with intragenic 5hmC (log2 CMS-IP/Input > 2) in at least one of the two cell types were considered and genes with an absolute difference of >0.5 between them were plotted. Number of genes in each quadrant is shown and depicted genes are listed in Supplementary Data 2. **c,** Venn diagram showing the number of genes which display novel chromatin accessibility peaks upon TCR activation among the group of 350 genes that are upregulated during CD4SP differentiation in the thymus (a fold change >2 in gene expression from DN3 to $CD4^{+}$SP) and undergo DNA demethylation (Intragenic 5hmC log2 CMS-IP/Input >2). **d,** Representative picture showcasing the lack of splenomegaly and lympho-adenopathy in *Rorc(t)Cre*^{Tg} *Tet1*$^{fl/fl}$ *Tet2*$^{fl/+}$ *Tet3*$^{fl/fl}$ mice (Top panel) versus severe splenomegaly and lympho-adenopathy in 5 weeks old *Rorc(t)Cre*^{Tg} *Tet1*$^{fl/fl}$ *Tet2*$^{fl/fl}$ Tet3$^{fl/fl}$ mice (Bottom). **e,** Representative FACS contour plots depicting proportions of CD4 and CD8 T cells among TCRβ^{+} T cells (Top panel) and proportions of naïve $CD4^{+}$ $CD62L^{hi}$ $CD44^{-}$ T cells among $CD4^{+}$ TCRβ^{+} T cells (Bottom Panel) in the spleen and LN of 5-6 weeks old control and *Rorc(t)Cre*^{Tg} *Tet1*$^{fl/fl}$ *Tet2*$^{fl/+}$ *Tet3*$^{fl/fl}$ mice. **f,** Histogram overlay of CD4, ThPOK, CD5 and CD6 expression on peripheral $CD4^{+}$ TCRβ^{+} T cells from the LN of *Rorc(t)*^{CreTg} *Tet1*$^{fl/fl}$ *Tet2*$^{fl/+}$ *Tet3*$^{fl/fl}$ mice (**top**) and FACS-sorted $CD4^{+}$ TCRβ^{+} T cells activated *in vitro* for 72hrs with anti-CD3/CD28

(bottom). Data is representative of >3 independent experiments. **g**, % of cells with hi or low gene expression per cell division cycle following *in vitro* activation for 72hrs with anti-CD3/CD28 (n=4 or 5/genotype). Data shown is mean ± SEM. ns=not significant (p=0.967 Top left, p>0.9999, p=0.3731 Top right, p>0.999 Bottom left, p=0.2564 Bottom right) ****p<0.0001 (Two-Way ANOVA with Bonferroni multiple comparison test). **h,** Normalized CD4 MFI expression in CD4 T cells from $Rag^{-/-}$ mice at day 7 post transfer. Naïve FACS-sorted WT CD45.1 and $Rorc(t)^{CreTg}$ $Tet1^{fl/fl}$ $Tet2^{fl/+}$ $Tet3^{fl/fl}$ mice CD45.2 T cells were transferred at a 1:1 ratio and transferred into $Rag^{-/-}$. Expression of the respective protein was normalized to the WT control (n=3) and data is representative of 2 independent experiments. Data shown is mean ± SEM.***p=0.0003, ****p<0.0001 (Two-Way ANOVA with Sidak's multiple comparison test).

Supplementary Table 1

Oligonucleotides sequences

Gene	Sequence
Cd4 promoter-F	AATGCCTGAGCAGAATCAAGCTG
Cd4 promoter-R	TAAGCCTTGCCTCACGTTGACCTA
Cd4 exon1-F	AGTTGAACACGTCCCTCTTACCTC
Cd4 exon1-R	GTTTGCAAAGTCTCGAGCCCTCAT
Cd4 S4 Silencer-F	TACGAAGCTAGGCAACAGAGGAAG
Cd4 S4 Silencer-R	TGTGGTCCCGAATGCGTTT
Cd4 E4p proximal enhancer-F	TCTCCAAAGGGTAACAGGTGTCAG
Cd4 E4p proximal enhancer-R	TGTGACTTACAAAGGCTGCCTCCA
Cd4 E4a enhanver-F	GCCCTTTGGGTTTGTGAGAG
Cd4 E4a enhancer-R	CACAAACCCAAAGGGCATGG
Cd4 E4m enhancer-F	GCC AAA GCA CAA GCA TGG AA
Cd4 E4m enhancer-R	GTC ACC CCT GGG AAA GGA TG
Cd5 mRNA-F	CACGGAGATCCTTGGCAGAA
Cd5 mRNA-R	TGAGATGTGGTGACCCCTTG
Cd6 mRNA-F	AATTGGCTCCCCTGCATCTC
Cd6 mRNA-R	CACCTCTGAGCTTCCACCAG
Zbtb7b mRNA-F	TCC GCT TCA CCA GGA ATG AC
Zbtb7b mRNA-R	GTA GCT ATG CAG GAA GCG GG
Hprt mRNA-F	GTTGGGCTTACCTCACTGCT
Hprt mRNA-R	TCATCGCTAATCACGACGCT
Cd4 Ex2/3 mRNA-F	TGCCGAGCCATCTCTCTTAG
Cd4 Ex2/3 mRNA-R	CACCAGCGTCTTCCCTTGAG
Cd4 Ex1/2 mRNA-F	CCAGAGGCTCAGATTCCCAAC
Cd4 Ex1/2 mRNA-R	GCCTAAGAGAGATGGCTCGGC
Cd4 Ex3/4 mRNA-F	TGTCACTCAAGGGAAGACGC
Cd4 Ex3/4 mRNA-R	CGAAGGCGAACCTCCTCTAA
