# Supplementary material for: CD4 expression in effector T cells depends on DNA demethylation over a developmentally established stimulus-responsive element
Source: Nat Commun. 2022 Mar 18;13:1477. doi: 10.1038/s41467-022-28914-4 (PMC8933563; doi:10.1038/s41467-022-28914-4)
Supplement: Supplementary file 3 — Description of Additional Supplementary Files [file 41467_2022_28914_MOESM3_ESM.pdf]

Description of Additional Supplementary Files

File Name: Supplementary Data 1
Description: List of genes present in each quadrant of the scatter plot of Supplementary Figure 7a, showing a fold change >2 in gene expression from DN3 to CD4+ thymic T cell differentiation (y-axis) versus presence of 5hmC in CD4+ T cells (log2 CMS-IP/Input >2).

File Name: Supplementary Data 2
Description: List of genes in each quadrant of the scatter plot shown in Supplementary Figure 7b, depicting change in gene expression from DN3 to CD4+ thymic T cell differentiation ((log2FoldChange) > 1) versus change in intragenic 5hmC in CD4+ compared to CD4+CD8+ DP thymic T cells. Genes with intragenic 5hmC (log2 CMS-IP/Input > 2) in at least one of the two cell types were considered and genes with an absolute difference of >0.5 between them were plotted.

File Name: Supplementary Data 3
Description: List of genes which a) upregulated gene expression from DN3 to CD4 SP T cells (fold change > 2) b) exhibited 5hmC in DP or CD4 SP thymic T cells c) had open chromatin peaks and concomitant H3K27Ac marks present in their gene bodies or upstream of their annotated TSS and d) intersecting between a-c.

File Name: Supplementary Data 4
Description: List of genes belonging to datasets depicted in the Venn diagram of Supplementary Fig 7c, showing the number of genes which display novel chromatin accessibility peaks upon TCR activation among the group of 350 genes that are upregulated during CD4SP differentiation in the thymus (a fold change >2 in gene expression from DN3 to CD4+SP) and undergo DNA demethylation (Intragenic 5hmC log2 CMS-IP/Input >2)
