# Supplementary material for: CD4 expression in effector T cells depends on DNA demethylation over a developmentally established stimulus-responsive element
Source: Nat Commun. 2022 Mar 18;13:1477. doi: 10.1038/s41467-022-28914-4 (PMC8933563; doi:10.1038/s41467-022-28914-4)
Supplement: Supplementary file 8 — Reporting Summary [file 41467_2022_28914_MOESM8_ESM.pdf]

nature research

Corresponding author(s): Issuree Priya D.

Last updated by author(s): 2021/11/20

Reporting Summary

Nature Research wishes to improve the reproducibility of the work that we publish. This form provides structure for consistency and transparency in reporting. For further information on Nature Research policies, see our Editorial Policies and the Editorial Policy Checklist.

Statistics

For all statistical analyses, confirm that the following items are present in the figure legend, table legend, main text, or Methods section.

n/a	Confirmed	
☐	☒	The exact sample size (*n*) for each experimental group/condition, given as a discrete number and unit of measurement
☒	☐	A statement on whether measurements were taken from distinct samples or whether the same sample was measured repeatedly
☐	☒	The statistical test(s) used AND whether they are one- or two-sided<br>*Only common tests should be described solely by name; describe more complex techniques in the Methods section.*
☐	☒	A description of all covariates tested
☐	☒	A description of any assumptions or corrections, such as tests of normality and adjustment for multiple comparisons
☐	☒	A full description of the statistical parameters including central tendency (e.g. means) or other basic estimates (e.g. regression coefficient) AND variation (e.g. standard deviation) or associated estimates of uncertainty (e.g. confidence intervals)
☐	☒	For null hypothesis testing, the test statistic (e.g. *F*, *t*, *r*) with confidence intervals, effect sizes, degrees of freedom and *P* value noted<br>*Give P values as exact values whenever suitable.*
☒	☐	For Bayesian analysis, information on the choice of priors and Markov chain Monte Carlo settings
☒	☐	For hierarchical and complex designs, identification of the appropriate level for tests and full reporting of outcomes
☒	☐	Estimates of effect sizes (e.g. Cohen's *d*, Pearson's *r*), indicating how they were calculated

Our web collection on statistics for biologists contains articles on many of the points above.

Software and code

Policy information about availability of computer code

Data collection	BioRad Bio-Plex (Luminex 200) instrument was used for multiplex cytokine assays. A CytoFlex B5-R3-V5 (Beckman Coulter) or BD LSRII (BD Biosciences) was used to acquire flow cytometry data. Diva software was used to gate on sorted T cell populations on the AriaII (BD Biosciences) cell sorter.
Data analysis	Flow jo (v9.9.6,Tree Star) was used for all flow cytometry analysis. Prism 8 (v8.0)or Prism 9 (v9.0) was used to generate all graphs and statistics. DESeq2 was used to analyze RNASeq gene expression data.

For manuscripts utilizing custom algorithms or software that are central to the research but not yet described in published literature, software must be made available to editors and reviewers. We strongly encourage code deposition in a community repository (e.g. GitHub). See the Nature Research guidelines for submitting code & software for further information.

Data

Policy information about availability of data

All manuscripts must include a data availability statement. This statement should provide the following information, where applicable:

- Accession codes, unique identifiers, or web links for publicly available datasets
- A list of figures that have associated raw data
- A description of any restrictions on data availability

All new genomic datasets in this manuscript has been deposited in the Sequence Read Archive (SRA) with accession codes PNJNA765040 . Raw data associated with figures generated in this manuscript has been provided as a source file in this manuscript. Additional information is available upon reasonable demand from the corresponding author. Novel mouse lines and reagents generated in this manuscript may be directly requested from the corresponding author will be shared in accordance with a Materials Transfer Agreement policy at the University of Iowa.

Field-specific reporting

Please select the one below that is the best fit for your research. If you are not sure, read the appropriate sections before making your selection.

☒ Life sciences ☐ Behavioural & social sciences ☐ Ecological, evolutionary & environmental sciences

For a reference copy of the document with all sections, see nature.com/documents/nr-reporting-summary-flat.pdf

Life sciences study design

All studies must disclose on these points even when the disclosure is negative.

Sample size	Animal sample size estimates were determined using power analysis (power=90% and alpha=0.05) based on the mean and standard deviation from our previous studies and/or pilot studies using 4-5 animals per group for Leishmaniasis and LCMV experiments. No sample size calculations were performed for other experiments. Sample sizes were determined based on prior studies and experience. (Sellars et al. 2015, Issuree et al., 2018)
Data exclusions	No samples were excluded from analysis unless a) low cell viability or poor sample quality was an issue during data acquisition. Such samples were excluded prior to analysis (e.g. Fig 4D, Sup Fig3E) b) poor infections / lack of swelling were noted due to improper leishmania footpad injections. A note was made during the experiment and confirmed upon euthanasia of the animals and low detection of CD11ahi T cells in the draining lymph nodes of mice.
Replication	All experiments performed in this manuscript were repeated independently at least 2 times with multiple animals or biological samples per repeat. All attempts at replication were successful.
Randomization	For Leishmaniasis experiments, control and mutant mice were bred and housed in the same facility and experiments were performed by ensuring grouping by gender. No differences in outcomes were seen in male or female cohorts. Animals were assigned to experimental and control groups randomly. For phenotyping of T cell populations in the thymus, mice were grouped based on gender to reduce variability associated with differences in rate of thymic atrophies.
Blinding	Footpad injections and pathology analysis of Leishmania-infected animals were done in a single-blinded manner. Parasitic burdens by serial dilutions were assessed in a single-blinded manner. LCMV infections in mice were single-blinded. All other animal studies were not blinded as non-subjective means of quantification were used (i.e frequencies of cells, mean fluorescence intensities, CT values).

Reporting for specific materials, systems and methods

We require information from authors about some types of materials, experimental systems and methods used in many studies. Here, indicate whether each material, system or method listed is relevant to your study. If you are not sure if a list item applies to your research, read the appropriate section before selecting a response.

Materials & experimental systems

n/a	Involved in the study	
☐	☒	Antibodies
☐	☒	Eukaryotic cell lines
☒	☐	Palaeontology and archaeology
☐	☒	Animals and other organisms
☒	☐	Human research participants
☒	☐	Clinical data
☒	☐	Dual use research of concern

Methods

n/a	Involved in the study	
☒	☐	ChIP-seq
☐	☒	Flow cytometry
☒	☐	MRI-based neuroimaging

Antibodies

Antibodies used	anti-CD62L FITC Clone MEL-14 Tonbo Cat no. 35-0621-U500<br>anti-CD25 PE Clone PC61.5 Tonbo Cat no. 50-0251-U500<br>anti-CD44 APC Clone IM7 Tonbo Cat no. 20-0441-U100<br>anti-CD44 BV510 Clone IM7 Biolegend Cat no. 103044<br>anti-CD19 PerCp 5.5 Clone ID3 Tonbo Cat no. 65-0193-U100<br>anti-CD5 BV510 Clone 53-7.3 Biolegend Cat no. 100627<br>anti-CD6 PE Clone OX-129 Biolegend Cat no. 146404<br>anti-Zbtb7b Clone T43-94 BD Biosciences Cat no. 565500<br>anti-CD4 E450 Clone RM4-5 Tonbo Cat no. 75-0042-U100<br>anti-CD4 PECy7 Clone RM4-5 Tonbo Cat no. 60-0042-U100<br>anti-CD8a PECy7 Clone 53-6.7 Tonbo Cat no. 60-0081-U100<br>anti-CD8a APC Clone 53-6.7 Tonbo Cat no. 20-0081-U100<br>anti-CD8a APC-Cy7 Clone 53-6.7 Tonbo Cat no. 25-0081-U100

anti-Nur77 PE Clone 12.14 Thermo Fisher Scientific Cat no. 12-5965-82
anti-CD11a FITC Clone M17/4 Biolegend Cat no. 101106
anti-CD49d APC Clone R1-2 Biolegend Cat no. 103622
anti-TNF BV421 Clone MP6-XT22 Biolegend Cat no. 506318
anti-IFNg APC Clone XMG1.2 Tonbo Cat no. 20-7311-U100
anti-CD45.2 A700 Clone 104 Tonbo Cat no. 80-0454-U100
anti-CD45.1 PE Clone A20 Tonbo Cat no. 50-0453-U100
anti-GATA3 FITC Clone TWAJ Thermo Fisher Scientific Cat no. 53-9966-42
anti-Tbet PECy7 Clone 4B10 Thermo Fisher Scientific Cat no. 25-5825-82
Ghost Dye APC-Cy7 Tonbo Cat no. 13-0865-T500
Ghost Dye BV510 Tonbo Cat no. 13-0870-T500
Rabbit anti-H3K4me3 Diagenode Lot no. A1052D
Rabbit anti-H3K9me3 Diagenode Lot no. A2217P
purified hamster anti-CD3 Clone 17A2 Tonbo Cat no. 70-0032-U500 or BioXcell Clone 17A2 Cat no. BE0002
purified hamster anti-CD28 Clone 37.51 Tonbo Cat no. 70-0281-U500 or BioXcell Clone 37.51 Cat no. BE0015-1
Goat Affinity Purified Antibody To Hamster IgG MP Biomedicals Cat no. 0856984

Validation

All antibodies were purchased from Tonbo, BioXcell, Biolegend, ThermoFisher and Diagenode. All commercial antibodies were validated by the supplier for their target and application, with relevant dilution, publications and validation information available on the manufacturer's websites for each antibody. All this information can be retrieved using the catalogue number listed above on the supplier's website.

Eukaryotic cell lines

Policy information about cell lines

Cell line source(s)	Plate-E Retroviral packaging cells were purchased from Cell Biolabs.
Authentication	Cell lines used behaved as expected and reported in the literature but were not authenticated.
Mycoplasma contamination	mycoplasma contamination was not tested
Commonly misidentified lines (See ICLAC register)	No commonly misidentified cell lines were used.

Animals and other organisms

Policy information about studies involving animals; ARRIVE guidelines recommended for reporting animal research

Laboratory animals	WT C57Bl/6J mice were obtained from Jackson Laboratories and bred in house. All other animals used in the study were generated or backcrossed onto C57Bl/6J background. Littermates with matched sex (both males and females) were used. Mice in all the experiments were 5-8 weeks old at the starting point of experiments. All mice for housed in specific pathogen free environment on a 12 hour light cycle at 30-70% humidity and temperature of 20-26 degrees celsius, with access to standard chow and water.
Wild animals	No wild animals were used in this study
Field-collected samples	No field collected samples were used in this study
Ethics oversight	All mice were maintained under specific pathogen-free conditions at the barrier animal facility at University of Iowa Carver College of Medicine. All experiments were performed in accordance with the protocol approved by the IACUC at the University of Iowa Carver College of Medicine.

Note that full information on the approval of the study protocol must also be provided in the manuscript.

Flow Cytometry

Plots

Confirm that:

☒ The axis labels state the marker and fluorochrome used (e.g. CD4-FITC).

☒ The axis scales are clearly visible. Include numbers along axes only for bottom left plot of group (a 'group' is an analysis of identical markers).

☒ All plots are contour plots with outliers or pseudocolor plots.

☒ A numerical value for number of cells or percentage (with statistics) is provided.

Methodology

Sample preparation

Cell fractions from the thymus, spleen and lymph nodes were obtained by mashing through a 70 micron strainer. Red blood cell lysis with ACK lysis buffer was performed. Cells were then stained with antibodies in 2% IMDM for 30 min at 4 degrees for flow cytometry. To obtained purified naive CD4 T cell populations, cells were first enriched using magnetic-activated cell sorting with untouched CD4 Dynabeads (Thermo Fisher Scientific) or the CD4+ T cell selection kit (Miltenyi), followed by

staining for Fluorescence-activated cell sorting on a Fusion or Aria Cell sorter based on expression of CD62L and CD44. Cells were sorted into 15mL conical tubes containing 500ul FBS.

For low input ChIP, cells were FACS-Sorted into 1.5mL tubes and used either immediately or flash frozen in liquid nitrogen and storage in the -80 for no longer than 2 weeks.

For methylation analysis by CATCH-Seq, sorted cell populations were collected for genomic DNA extraction using a DNA extraction kit and DNA was stored at -80 until ready for shearing, hybridization and bisulfite treatment.
For RNA extraction, cells were lysed in RLT lysis buffer immediately after cell sorting and stored at -80 until ready for extraction.

Retroviruses were prepared by transfection of Plate-E cells and collection of cell supernatants 3 days post transfection. Supernatants were used right away or within a week, stored at 4 degrees.

For assessment of cytokines and parasite burden in the footpad of Leishmania- infected mice, footpads were surgically dissected and homogenized by meticulous douncing. Samples were spun at 500g for 10 min to pellet cellular debris. Supernatants were collected for immediate analysis of parasitic load by serial dilution or frozen at -80 for later measurements of cytokines using the BioPlex assay.

Splenocytes from LCMV-infected mice were isolated using Collagenase D digestion and were incubated with indicated doses of the LCMV-derived peptide GP66–80 in the presence of 3 µg/ml brefeldin A for cytokine assessment by flow cytometry.

Instrument

LSRII or AriaII (BD Biosciences) or Cytoflex (BC Life sciences)

Software

Diva (BD Biosciences) and Flowjo 9.9.6 (Tree Star)

Cell population abundance

The purities of sorted T cells were more than 98%

Gating strategy

Based on the pattern of FSC-A/SSC-A, cells in the lymphocyte gate were used for analysis of T cell subsets. Singlets were gated according to the pattern of SSC-H vs. SSC-W. Ghost dyes were used to exclude non-viable cells in the analysis. In heterogenous cell fractions, positive and negative populations were determined by comparing staining on non-specific versus specific cell populations. Fluorescence minus one (FMO) controls were also included in some instances. In the case of purified cell populations, positive staining was always determined using FMO controls.

☒ Tick this box to confirm that a figure exemplifying the gating strategy is provided in the Supplementary Information.
